# Trajectories of Risk for Specific Readmission Diagnoses after Hospitalization for Heart Failure, Acute Myocardial Infarction, or Pneumonia

**DOI:** 10.1371/journal.pone.0160492

**Published:** 2016-10-07

**Authors:** Harlan M. Krumholz, Angela Hsieh, Rachel P. Dreyer, John Welsh, Nihar R. Desai, Kumar Dharmarajan

**Affiliations:** 1 Section of Cardiovascular Medicine, Department of Internal Medicine, Yale School of Medicine, New Haven, CT, United States of America; 2 Center for Outcomes Research and Evaluation, Yale-New Haven Hospital, New Haven, CT, United States of America; 3 Department of Health Policy and Management, Yale School of Public Health, New Haven, CT, United States of America; 4 Robert Wood Johnson Foundation Clinical Scholars Program, Department of Internal Medicine, Yale School of Medicine, New Haven, CT, United States of America; Centre National de la Recherche Scientifique, FRANCE

## Abstract

**Background:**

The risk of rehospitalization is elevated in the immediate post-discharge period and declines over time. It is not known if the extent and timing of risk vary across readmission diagnoses, suggesting that recovery and vulnerability after discharge differ by physiologic system.

**Objective:**

We compared risk trajectories for major readmission diagnoses in the year after discharge among all Medicare fee-for-service beneficiaries hospitalized with heart failure (HF), acute myocardial infarction (AMI), or pneumonia from 2008–2010.

**Methods:**

We estimated the daily risk of rehospitalization for 12 major readmission diagnostic categories after accounting for the competing risk of death after discharge. For each diagnostic category, we identified (1) the time required for readmission risk to peak and then decline 50% from maximum values after discharge; (2) the time required for readmission risk to approach plateau periods of minimal day-to-day change; and (3) the extent to which hospitalization risks are higher among patients recently discharged from the hospital compared with the general elderly population.

**Results:**

Among >3,000,000 hospitalizations, the yearly rate of rehospitalization was 67.0%, 49.5%, and 55.3% after hospitalization for HF, AMI, and pneumonia, respectively. The extent and timing of risk varied by readmission diagnosis and initial admitting condition. Risk of readmission for gastrointestinal bleeding/anemia peaked particularly late after hospital discharge, occurring 10, 6, and 7 days after hospitalization for HF, AMI, and pneumonia, respectively. Risk of readmission for trauma/injury declined particularly slowly, requiring 38, 20, and 38 days to decline by 50% after hospitalization for HF, AMI, and pneumonia, respectively.

**Conclusions:**

Patterns of vulnerability to different conditions that cause rehospitalization vary by time after hospital discharge. This finding suggests that recovery of various physiologic systems occurs at different rates and that post-discharge interventions to minimize vulnerability to specific conditions should be tailored to their underlying risks.

## Introduction

Post-hospital syndrome describes a period of transient, generalized risk after hospital discharge.[1] The risks of readmission and death following hospitalization are markedly elevated in the immediate post-discharge period and slowly decline over time, with variation by admitting diagnosis and outcome.[2] During this period, patients remain vulnerable to illness that could lead to rehospitalization from a wide range of medical conditions beyond the reason for the initial hospitalization.[3, 4] For example, within 30-days of hospitalization, the proportion of patients readmitted for recurrent heart failure (HF), recurrent acute myocardial infarction (AMI), and recurrent pneumonia is only 35%, 10%, and 22% of all readmissions, respectively.[3]

A key unanswered question is whether the timing of vulnerability after hospital discharge varies for different medical conditions that commonly result in rehospitalization. For example, following hospitalization for HF, does the timing of risk for recurrent HF differ from that of infections? How does the timing of risk for bleeding compare with renal dysfunction? Is the period of elevated risk particularly prolonged for specific conditions that commonly result in readmission? Such information can be useful in understanding if recovery from acute illness varies by physiologic system. Findings can guide future basic and translational work to elucidate mechanisms responsible for potential differences in the extent and timing of risk across conditions. This information can also demonstrate if patients remain particularly vulnerable to specific conditions after the immediate post-discharge period when clinical follow up grows less regular. Interventions could ultimately be developed to support patients and mitigate these extended periods of vulnerability to specific conditions. However to date, we have no information about how patients’ susceptibility to specific causes of readmission varies over time.

Accordingly, we compared risk trajectories for major readmission diagnoses for the national population of Medicare fee-for-service beneficiaries initially hospitalized with HF, AMI, or pneumonia in the year after hospital discharge. These 3 conditions are among the most common reasons for hospitalization in older patients[5] and have been a focus of federal quality improvement efforts.[6–12] We classified readmission diagnoses into major diagnostic categories and determined the time that it takes for readmission risk to peak after discharge, decay by 50 percent, and achieve plateau periods of minimal day-to-day change. We also compared the magnitude and timing of readmission risk for each diagnostic category with the risk of hospital admission for these diagnoses in the general Medicare population.

## Methods

### Study Sample

We used Medicare Standard Analytic and Denominator files to identify hospitalizations at acute care hospitals from 2008 to 2010 with a principal discharge diagnosis of HF, AMI, or pneumonia. Cohorts were defined with International Classification of Diseases, Ninth Revision, Clinical Modification (ICD-9-CM) codes identical to those used in the publicly reported readmission and mortality measures of the Centers for Medicare & Medicaid Services (CMS) (S1 Table).[8, 10, 12] We included hospitalizations among patients aged 65 years or older. We excluded patients with in-hospital death, those who were enrolled in Medicare fee-for-service for less than 1-year post-discharge in the absence of death, those who were transferred to another acute care facility, and those who were discharged against medical advice. Similar to the CMS measures, our analyses of rehospitalization used all index hospitalizations across the 3 study years. Our analyses of death used 1 random hospitalization per patient over the 3-year period.

### Sample Classification

We categorized readmission diagnoses using a modified version of the CMS Condition Categories (CC’s) as has been done previously.[13, 14] We further consolidated the group of 30 modified CCs, or readmission diagnostic categories, into 12 clinically meaningful domains to facilitate data presentation. For instance, readmission diagnostic categories related to AMI, unstable angina, and other acute ischemic heart disease, as well as acute stroke and transient ischemic attack, constituted the category ‘cardiovascular disease (CVD).’ The specific diagnoses comprising each readmission diagnostic category are presented in S2 Table. Similar approaches to aggregating readmission diagnoses have been used previously.[3, 4]

### Rehospitalizations

We classified all first rehospitalizations into one of 12 diagnostic categories as above and calculated the daily risk of rehospitalization due to each in the year after hospital discharge. As with the CMS measures, we only included readmissions to short-term acute care hospitals and excluded rehospitalizations that were considered planned based upon the presence of specific ICD-9-CM procedure and principal discharge diagnosis codes.[8, 10, 12] In parallel with the CMS measures, we did not consider transfers to other hospitals on the day of discharge or the day after discharge to be rehospitalizations. We classified the rehospitalizations by their principal discharge diagnoses.

### Medicare Comparison Population

We employed the method to compare risks of readmission for major diagnostic categories following hospitalization for HF, AMI, or pneumonia with the risks of hospitalization for these same diagnoses in the overall Medicare population that has been used previously.[2] We used the 2009 Medicare Provider Analysis and Review and Denominator files. We included all Medicare fee-for-service beneficiaries who were aged 65 years or older on January 1, 2009 and had at least 12 months of enrollment in fee-for-service Medicare in the absence of death.

### Statistical Analyses

#### Daily risk of rehospitalization for specific readmission diagnostic categories

We fit separate survival models for the daily risk of rehospitalization for each specific diagnostic category. We considered death prior to rehospitalization and rehospitalization for other diagnosis categories to be competing risks that preclude rehospitalization for the specific diagnosis of interest. For example, following hospitalization for AMI, both death and readmission for all diagnoses besides HF compete with readmission for HF. To account for these competing risks in our survival models, we calculated the subdistribution hazard for first rehospitalization for each diagnostic category. These subdistribution hazards were derived from the cumulative incidence function for each day (1–365) after hospital discharge using the approach by Fine and Gray.[15] This approach provides estimations of unconditional risk after consideration of competing risks by keeping competing risk observations in the risk set for survival analysis, but with a diminished weight. We censored data at planned readmissions or 1 year following index hospitalization, whichever occurred first.

To characterize changes in rehospitalization risk due to each diagnostic category over time, we identified the time required for the daily risk of each to decline by 50% by using the bootstrap method with 2,000 iterations to construct 95% confidence intervals (CIs) for the time required for the risks of readmission for each diagnostic category to decline 50% from its maximum subdistribution hazard after discharge. The bootstrap method permits robust non-parametric estimates of confidence intervals when the estimated statistic is not asymptotically normally distributed.[16] The method involves sampling of the original dataset with replacement to generate a new dataset that is the same size as the original. Statistical analyses are repeated on each of these samples to generate 95% confidence intervals.

To identify the time after which readmission risk for each diagnostic category is nearly constant with minimal day-to-day change, we first calculated the change in daily risk of rehospitalization due to each readmission diagnostic category as the difference in kernel-smoothed subdistribution hazard estimates between each day and the preceding day. For each day after maximum hazard, we divided the change in daily risk by its maximum daily decline after discharge. We then identified the time required for the change in daily risk to decline 95% from its maximum daily decline after discharge. This time point denotes a period after which risk for each readmission diagnostic category is largely invariant and potentially associated with a new stage of recovery. We used the bootstrap method with 2,000 iterations to construct associated 95% CIs.

#### Relative risks of hospitalization for each readmission diagnostic category in the study populations compared with the Medicare fee-for-service population

We first calculated the 1-year incidence of hospitalization for each of the 12 major diagnostic categories among all Medicare fee-for-service beneficiaries in 2009. We then prorated these 1-year incidence rates over days to calculate the cumulative incidence of rehospitalization for each major diagnostic category by day after discharge (1–365) following the index hospitalization for HF, AMI, or pneumonia.

To make study and comparator populations more similar in age, sex, race, and income, we used direct standardization to calculate the age-sex-race-income standardized cumulative incidence of hospitalization for each of the 12 major diagnostic categories in the 3 study cohorts and in the Medicare fee-for-service population. The 2009 Medicare fee-for-service population was used as the standard population. We used 3 age categories (65–74 years, 75–84 years, and ≥85 years), 2 sex categories, 3 race categories (white, black, other), and 3 income groups (low: below or equal to the 3^rd^ decile of median zip code income; median: above the 3^rd^ decile and below the 7^th^ decile of median zip code income; high: above the 7^th^ decile of median zip code income). We then calculated the relative risks of hospitalization for each diagnostic category between study cohorts and the Medicare fee-for-service population over the first 30, 60, 90, 180, and 365 days after discharge and used the bootstrap method to construct the 95% CIs of the relative risk.

Analyses were primarily conducted using SAS 9.3 (SAS Institute, Cary, North Carolina) by AFH. We obtained Institutional Review Board approval, including waiver of the requirement for participant informed consent, from the Yale University Human Investigation Committee.

## Results

We identified 1,922,580 hospitalizations for HF from 4,764 hospitals, 742,335 hospitalizations for AMI from 4,502 hospitals, and 1,497,271 hospitalizations for pneumonia from 4,795 hospitals. These cohorts were comprised of 1,230,841, 674,799 and 1,236,060 unique patients, respectively. The Medicare fee-for-service comparison population consisted of 27,764,699 persons. The demographic characteristics of these 4 populations are shown in S3 Table.

Within 1 year of discharge, rehospitalization occurred after 67.0% of HF hospitalizations, 49.5% of AMI hospitalizations, and 55.3% of pneumonia hospitalizations. The demographic characteristics of the people who were rehospitalized, stratified by readmission diagnostic category, are described in Table 1.

**Table 1:** Demographic Characteristics of Rehospitalizations by Readmission Diagnostic Categories.

**(A) Heart Failure**						
**Readmission Diagnosis**	**N (% of All Readmissions)**	**Mean Age in Years (SD)**	**Female (%)**	**Race**		
				**White (%)**	**Black (%)**	**Other (%)**
**Cardiovascular Disease**	61,765 (4.8)	81.3 (8.3)	41.8%	81.3%	13.5%	5.2%
**Heart Failure**	459,587 (35.7)	80.2 (8.2)	45.9%	79.5%	15.6%	4.9%
**Stable Coronary Artery Disease/Angina/Chest Pain**	10,601 (0.8)	79.0 (8.0)	49.7%	82.7%	11.6%	5.7%
**Pulmonary Embolism/Deep Vein Thrombosis**	8,101 (0.6)	80.8 (8.2)	39.4%	77.3%	19.1%	3.6%
**Chronic Obstructive Pulmonary Disease/Asthma**	50,730 (3.9)	78.3 (7.8)	43.0%	83.3%	12.6%	4.1%
**Other Cardiopulmonary**	117,094 (9.1)	79.6 (8.1)	42.9%	80.7%	14.4%	4.9%
**Gastrointestinal Bleeding/Anemia**	23,543 (1.8)	80.7 (7.9)	42.2%	82.6%	12.7%	4.8%
**Infection**	177,085 (13.8)	81.4 (8.1)	42.0%	84.6%	10.6%	4.8%
**Trauma/Injury**	43,024 (3.3)	83.2 (7.6)	33.4%	91.7%	4.9%	3.4%
**Renal/Metabolic Disorders**	116,859 (9.1)	79.5 (8.1)	44.1%	76.8%	17.6%	5.6%
**Arrhythmia/Conduction Disorders**	43,485 (3.4)	80.1 (8.0)	40.9%	85.0%	11.0%	4.0%
**Other**	175,634 (13.6)	79.8 (8.0)	45.3%	81.4%	14.0%	4.6%
**(B) Acute Myocardial Infarction**						
**Readmission Diagnosis**	**N (% of All Readmissions)**	**Mean Age in Years (SD)**	**Female (%)**	**Race**		
				**White (%)**	**Black (%)**	**Other (%)**
**Cardiovascular Disease**	54,275 (14.8)	80.1 (8.5)	51.4%	85.8%	9.3%	4.9%
**Heart Failure**	61,220 (16.7)	80.4 (8.3)	53.2%	85.1%	9.9%	5.0%
**Stable Coronary Artery Disease/Angina/Chest Pain**	12,582 (3.4)	78.1 (8.1)	48.1%	87.0%	8.3%	4.7%
**Pulmonary Embolism/Deep Vein Thrombosis**	4,293 (1.2)	78.9 (8.0)	55.2%	84.5%	12.5%	3.1%
**Chronic Obstructive Pulmonary Disease/Asthma**	11,288 (3.1)	77.1 (7.6)	54.0%	88.2%	8.0%	3.8%
**Other Cardiopulmonary**	42,898 (11.7)	78.1 (8.1)	53.6%	85.4%	9.8%	4.8%
**Gastrointestinal Bleeding/Anemia**	9,173 (2.5)	79.7 (8.1)	52.7%	86.5%	8.8%	4.7%
**Infection**	50,793 (13.8)	80.6 (8.3)	53.5%	86.3%	8.5%	5.2%
**Trauma/Injury**	13,071 (3.6)	82.4 (7.8)	65.4%	92.7%	3.6%	3.7%
**Renal/Metabolic Disorders**	24,660 (6.7)	79.3 (8.2)	54.1%	80.0%	13.5%	6.4%
**Arrhythmia/Conduction Disorders**	15,668 (4.3)	79.2 (8.0)	52.3%	89.5%	6.7%	3.8%
**Other**	67,343 (18.3)	78.5 (8.0)	50.6%	85.7%	9.7%	4.6%
**(C) Pneumonia**						
**Readmission Diagnosis**	**N (% of All Readmissions)**	**Mean Age in Years (SD)**	**Female (%)**	**Race**		
				**White (%)**	**Black (%)**	**Other (%)**
**Cardiovascular Disease**	31,581 (3.8)	81.6 (8.0)	55.6%	88.0%	7.5%	4.5%
**Heart Failure**	67,488 (8.2)	81.6 (8.1)	54.4%	87.3%	8.4%	4.3%
**Stable Coronary Artery Disease/Angina/Chest Pain**	3,726 (0.5)	79.2 (8.0)	44.9%	87.6%	7.4%	5.1%
**Pulmonary Embolism/Deep Vein Thrombosis**	10,771 (1.3)	80.3 (7.9)	57.4%	87.1%	9.7%	3.2%
**Chronic Obstructive Pulmonary Disease/Asthma**	79,636 (9.6)	77.6 (7.5)	54.2%	90.1%	5.7%	4.2%
**Other Cardiopulmonary**	89,071 (10.8)	79.0 (7.9)	54.7%	87.1%	8.1%	4.7%
**Gastrointestinal Bleeding/Anemia**	13,963 (1.7)	80.8 (8.0)	54.4%	85.7%	8.9%	5.4%
**Infection**	285,909 (34.6)	80.7 (8.2)	52.7%	93.3%	2.9%	3.8%
**Trauma/Injury**	36,985 (4.5)	82.8 (7.8)	65.7%	82.0%	12.1%	6.0%
**Renal/Metabolic Disorders**	49,857 (6.0)	80.3 (8.2)	56.2%	90.6%	5.7%	3.8%
**Arrhythmia/Conduction Disorders**	20,644 (2.5)	80.8 (7.8)	56.7%	90.5%	5.7%	3.8%
**Other**	137,812 (16.7)	79.6 (8.0)	53.7%	86.5%	8.7%	4.8%

SD, standard deviation

**Fig 1:**
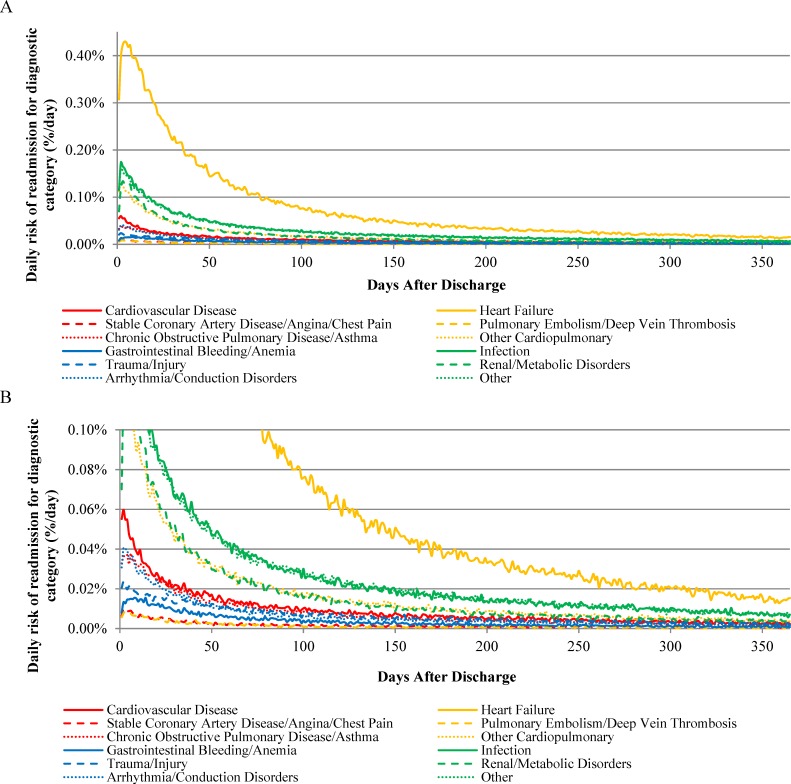
Daily Risk of Rehospitalization for Common Diagnostic Categories After Hospitalization for Heart Failure.

**Fig 2:**
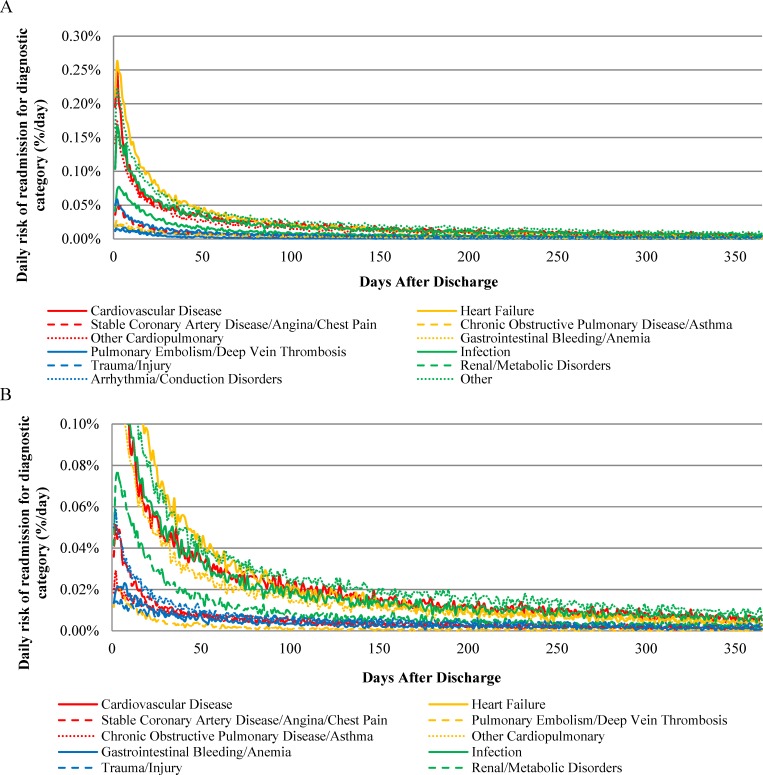
Daily Risk of Rehospitalization for Common Diagnostic Categories After Hospitalization for Acute Myocardial Infarction.

**Fig 3:**
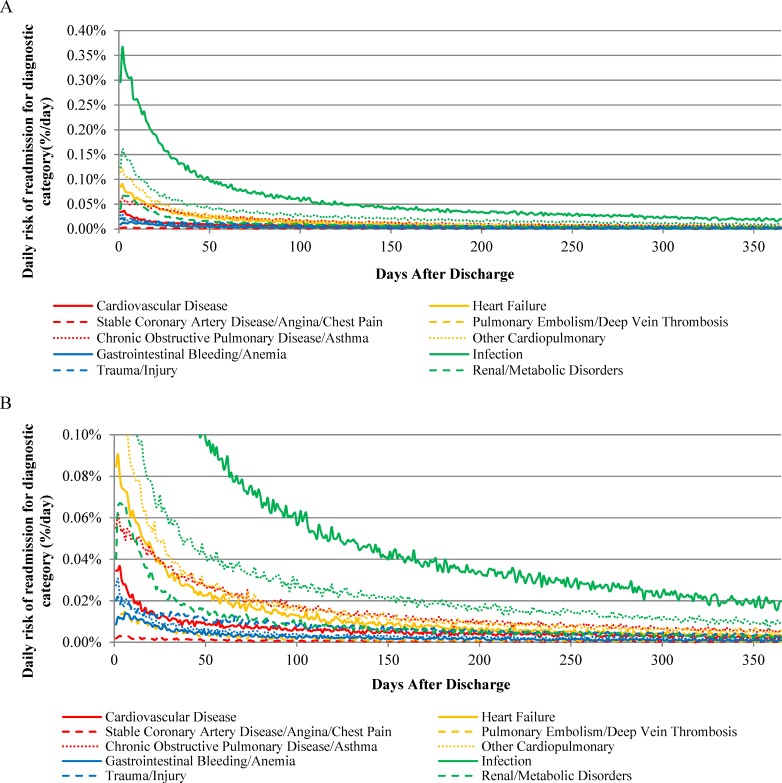
Daily Risk of Rehospitalization for Common Diagnostic Categories After Hospitalization for Pneumonia.

The absolute risk of rehospitalization varied across readmission diagnostic categories following hospitalization for HF, AMI, or pneumonia (Figs 1, 2 and 3, respectively). In addition, the day of peak rehospitalization risk after hospital discharge varied across readmission diagnostic categories (Table 2). For example, after HF hospitalization, the daily risk of readmission for HF peaked on day 4 and the daily risk of readmission for gastrointestinal bleeding/anemia peaked on day 10. After AMI hospitalization, the daily risk of readmission for CVD peaked on day 2 and the daily risk of readmission for gastrointestinal bleeding/anemia peaked on day 6. After pneumonia hospitalization, the daily risk of readmission for infection peaked on day 2 and the daily risk of readmission for gastrointestinal bleeding/anemia peaked on day 7.

**Table 2:** Representative Time Points Describing Trajectories of Risk for Rehospitalization for Readmission Diagnostic Categories.

Admitting Diagnosis	Readmission Diagnosis	Day of Highest Risk	Days for the Level of Risk to Decline 50%	Days for the Daily Change in Risk to Decline 95%
**Heart Failure**				
	Cardiovascular Disease	2	16 (15–18)	31 (27–33)
	Heart Failure	4	32 (30–36)	50 (40–52)
	Stable Coronary Artery Disease/Angina/Chest Pain	2	17 (10–24)	32 (17–43)
	Pulmonary Embolism/Deep Vein Thrombosis	3	18 (16–24)	37 (19–51)
	Chronic Obstructive Pulmonary Disease/Asthma	5	29 (27–32)	41 (27–54)
	Other Cardiopulmonary	2	18 (16–21)	38 (32–40)
	Gastrointestinal Bleeding/Anemia	10	33 (30–40)	40 (19–54)
	Infection	2	21 (21–23)	41 (32–46)
	Trauma/Injury	2	38 (29–49)	39 (15–56)
	Renal/Metabolic Disorders	3	20 (20–22)	43 (39–46)
	Arrhythmia/Conduction Disorders	2	22 (19–25)	33 (20–39)
	Other	2	23 (22–24)	44 (39–47)
**Acute Myocardial Infarction**				
	Cardiovascular Disease	2	7 (6–8)	21 (21–22)
	Heart Failure	2	12 (12–13)	33 (33–36)
	Stable Coronary Artery Disease/Angina/Chest Pain	2	10 (10–11)	26 (20–30)
	Pulmonary Embolism/Deep Vein Thrombosis	4	13 (9–15)	29 (22–34)
	Chronic Obstructive Pulmonary Disease/Asthma	2	10 (6–16)	25 (11–33)
	Other Cardiopulmonary	2	10 (9–11)	28 (24–30)
	Gastrointestinal Bleeding/Anemia	6	18 (15–22)	32 (18–41)
	Infection	2	14 (13–15)	35 (31–40)
	Trauma/Injury	5	20 (13–26)	44 (20–56)
	Renal/Metabolic Disorders	3	17 (15–19)	39 (24–45)
	Arrhythmia/Conduction Disorders	2	10 (8–11)	25 (20–31)
	Other	2	13 (11–13)	29 (26–32)
**Pneumonia**				
	Cardiovascular Disease	3	12 (11–15)	27 (19–31)
	Heart Failure	2	20 (17–22)	40 (33–47)
	Stable Coronary Artery Disease/Angina/Chest Pain	3	12 (9–22)	48 (18–64)
	Pulmonary Embolism/Deep Vein Thrombosis	6	20 (18–23)	31 (15–42)
	Chronic Obstructive Pulmonary Disease/Asthma	2	36 (33–43)	32 (12–46)
	Other Cardiopulmonary	2	17 (16–18)	42 (39–46)
	Gastrointestinal Bleeding/Anemia	7	23 (21–26)	40 (25–51)
	Infection	2	21 (21–22)	51 (47–54)
	Trauma/Injury	3	38 (19–52)	33 (17–42)
	Renal/Metabolic Disorders	4	18 (17–19)	37 (30–39)
	Arrhythmia/Conduction Disorders	2	11 (5–14)	26 (19–30)
	Other	2	18 (17–21)	34 (26–39)

The time required for the daily risk of readmission to decline by 50 percent for each readmission diagnostic category is presented in Table 2. The daily risk of rehospitalization was 50% lower than the peak risk by 30 days for most diagnostic categories for patients initially hospitalized with HF, by 14 days for most diagnostic categories for patients initially hospitalized with AMI, and by 25 days for most diagnostic categories for patients initially hospitalized with pneumonia (Table 2). In all instances, however, the time required for the daily risk of readmission to decline by 50% was highest for the trauma/injury category, requiring 38, 20, and 38 days after hospitalization for HF, AMI, and pneumonia, respectively.

The daily risk of rehospitalization approached plateau periods of minimal day-to-day change by 60 days after hospitalization for all readmission diagnostic categories for all 3 index conditions (Table 2). Following hospitalization for HF, the number of days required for the daily change in risk of readmission for the 12 diagnostic categories to decline 95% from their maximum daily declines after discharge was 31–50 days: 31 days (95% CI 27–33 days) for CVD-related readmissions and 50 days (95% CI, 40–52 days) for HF readmissions. Following hospitalization for AMI, the number of days required for the daily change in risk of readmission for the 12 diagnostic categories to decline 95% from their maximum daily declines after discharge was 21–44 days: 21 days (95% CT 21–22 days) for CVD-related readmissions and 44 days (95% CI, 20–56 days) for trauma/injury readmissions. Following hospitalization for pneumonia, the number of days required for the daily change in risk of readmission for the 12 diagnostic categories to decline 95% from their maximum daily declines after discharge was 26–51 days: 26 days (95% CT 19–30 days) for arrhythmia/conduction disorder-related readmissions and 51 days (95% CI, 47–54 days) for infection-related readmissions.

The age-sex-race-income standardized relative risk of rehospitalization for each readmission diagnostic category was significantly higher than the baseline risk of hospitalization for the same diagnosis among the comparator population of Medicare fee-for-service beneficiaries (S1, S2 and S3 Figs). Relative risks comparing study populations to the Medicare fee-for-service population for all outcomes and conditions over the first 30, 60, 90, 180, and 365 days after discharge are presented in S4 Table.

## Discussion

With data from more than 3 million unique patients hospitalized for HF, AMI, or pneumonia, we show, for the first time, how the pattern of vulnerability to different conditions causing rehospitalization varies over time and by cause of the index hospitalization. This study extends our prior work showing that patients are susceptible to a broad range of medical conditions after hospitalization[3] by revealing that risks of rehospitalization for specific diagnoses have distinctive patterns for different physiological systems. For example, the risk of readmission for gastrointestinal bleeding and anemia peak especially late after hospital discharge while the risk of readmission for trauma and injury decline especially slowly. These heterogeneous patterns can guide future work to elucidate the biopsychosocial mechanisms responsible for differences in the extent and timing of risk across conditions. This information can also help align clinical follow up and surveillance after hospitalization to the specific conditions most likely to cause adverse outcomes.

A central observation is that vulnerability is not the same for all types of conditions that cause rehospitalization. Many conditions have risks that are greatest in the earliest period after discharge and then decline rapidly. However, several conditions have peak risks that occur later. For example, the risk of rehospitalization due to gastrointestinal bleeding peaks particularly late after hospitalization for all 3 index conditions and remains elevated for relatively longer periods of time. This finding may relate to the fact that stress from acute illness and hospitalization over time manifests as damage to the gastrointestinal tract. This finding may also relate to new treatments instituted during hospitalization, but since this pattern crosses conditions, it seems unlikely to be primarily related to the use of anti-platelet agents. This relatively prolonged risk for gastrointestinal bleeding also seems unlikely to be strictly due to impaired hemostasis since other types of bleeding that cause rehospitalization are not delayed. As a result, clinicians need to be vigilant for gastrointestinal bleeding well beyond the initial post-discharge outpatient visits that occur soon after discharge, as bleeding due to illness and treatment may not be appreciable until a relatively long time after hospitalization.

Our results also show that the risk of trauma and injury remains elevated for a particularly long time across conditions. Although the reason for this phenomenon is unknown, it raises questions about the mechanism by which these events transpire and may relate to patient strength, balance, and judgment. The prolonged period of risk may occur because many patients who initially receive support with activities and instrumental activities of daily living soon after hospitalization lose some of this assistance in subsequent weeks, leaving them more vulnerable to injury due to continued weakness, gait instability, or disorientation. It is also possible that patients themselves under-estimate their continued impairments after hospitalization and do not adequately protect themselves from injury. It may also track with activity, as the risk may increase as people become more active. Physicians do not commonly counsel patients about risk of injury after hospitalization, so knowledge of this vulnerability shown in our data serves as an opportunity to improve care and outcomes for older patients.

A larger message of our study is that patients experience an elevated risk for a wide range of medical problems throughout the post-discharge period. The fact that patients have an elevated risk for all readmission diagnostic categories for an extended time after hospitalization suggests that these are not unrelated hospitalizations, but rather connected with the acute illness and the hospitalization. This pattern is consistent with the post-hospital syndrome hypothesis that patients are returning home with a transient condition of generalized vulnerability that derives from their acute illness as well as their experience in the hospital.[1]

Our findings have additional implications for clinical practice and research. Many programs designed to reduce readmission risk have tended to focus on the cause of the initial hospitalization.[17–19] However, our results suggest that during the first 2 months after discharge, it is imperative to concentrate on risks associated with a broader range of conditions than the index diagnosis and to recognize that risk for various causes of readmission varies over time. Future basic, translational, and clinical research is needed to investigate the functioning of the various physiologic systems during hospitalization and the post-hospital period. This work will help determine the underlying causes of perturbation of these biological systems and differences in timing of risk across conditions causing readmission. The answer will likely be some combination of patients’ underlying morbidities, acute illness processes, the care provided, available supports after hospitalization, and access to health care.

This work has several potential limitations. The study focused on Medicare fee-for-service beneficiaries and the findings may not extend to younger populations. Nevertheless, other studies have shown that younger patients also experience a transient period of vulnerability to a wide range of causes of readmission.[20] For causes of readmissions, we relied on diagnostic codes, which have a reasonable sensitivity and specificity, but are not the same as adjudicated endpoints.[21, 22] The grouping of codes allows for a clinically coherent analysis, but may have also introduced some noise into our analysis. In addition, the particular groupings used have not been previously validated, although they were created using a similar approach employed in prior analyses.[3, 23] We used administrative data for our analyses and therefore did not have access to granular clinical information from the medical chart. However, this information is not particularly relevant for calculating differences in readmission risk by readmission diagnosis. We did not seek to identify clinical factors that are predictive of readmission. Finally, we assessed diagnoses associated with only first readmissions; the examination of subsequent readmissions could provide further insights.

## Conclusion

In conclusion, this study is the first to characterize the daily risk of readmission from specific diagnoses for the national population of Medicare fee-for-service beneficiaries hospitalized with common medical conditions. The magnitude and timing of risk varied by readmission diagnosis and the cause of initial hospitalization, suggesting that impairments of various physiological systems occur with variable recovery times among patients who are recently hospitalized. These findings additionally suggest the potential importance of tailoring post-discharge interventions to minimize vulnerability to specific conditions over time, though the post-discharge period is notable for elevated readmission risk from a wide range of medical conditions. The next step is to understand how best to mitigate these impairments and reduce the risk of adverse events after hospitalization.

## Supporting Information

S1 Fig Relative Risk of Hospitalization for Major Diagnostic Categories in Study Cohorts Compared with the Medicare Fee-For-Service Population. Relative risk information for HF. Each line represents 1 of the 12 readmission diagnostic categories.
(TIFF)

S2 Fig Relative Risk of Hospitalization for Major Diagnostic Categories in Study Cohorts Compared with the Medicare Fee-For-Service Population. Relative risk information for AMI. Each line represents 1 of the 12 readmission diagnostic categories.
(TIF)

S3 Fig Relative Risk of Hospitalization for Major Diagnostic Categories in Study Cohorts Compared with the Medicare Fee-For-Service Population for pneumonia. Each line represents 1 of the 12 readmission diagnostic categories.
(TIF)

S1 Table International Classification of Disease, Ninth Revision, Clinical Modification Codes Used to Define HF, AMI, and Pneumonia Cohorts. (DOCX)

S2 Table Readmission Diagnostic Category Constituents. (DOCX)

S3 Table Age, Race, and Sex Distribution for the 3 Condition Cohorts and Comparator Population. (DOCX)

S4 Table Relative Risks Comparing Study Populations to the Medicare Fee-For-Service Population for All Outcomes and Conditions Over the First 30, 60, 90, 180, and 365 Days After Discharge. (DOCX)
