# Supplementary figures and images for: Trajectories of Risk for Specific Readmission Diagnoses after Hospitalization for Heart Failure, Acute Myocardial Infarction, or Pneumonia
Source: PLoS One. 2016 Oct 7;11(10):e0160492. doi: 10.1371/journal.pone.0160492 (PMC5055318; doi:10.1371/journal.pone.0160492)

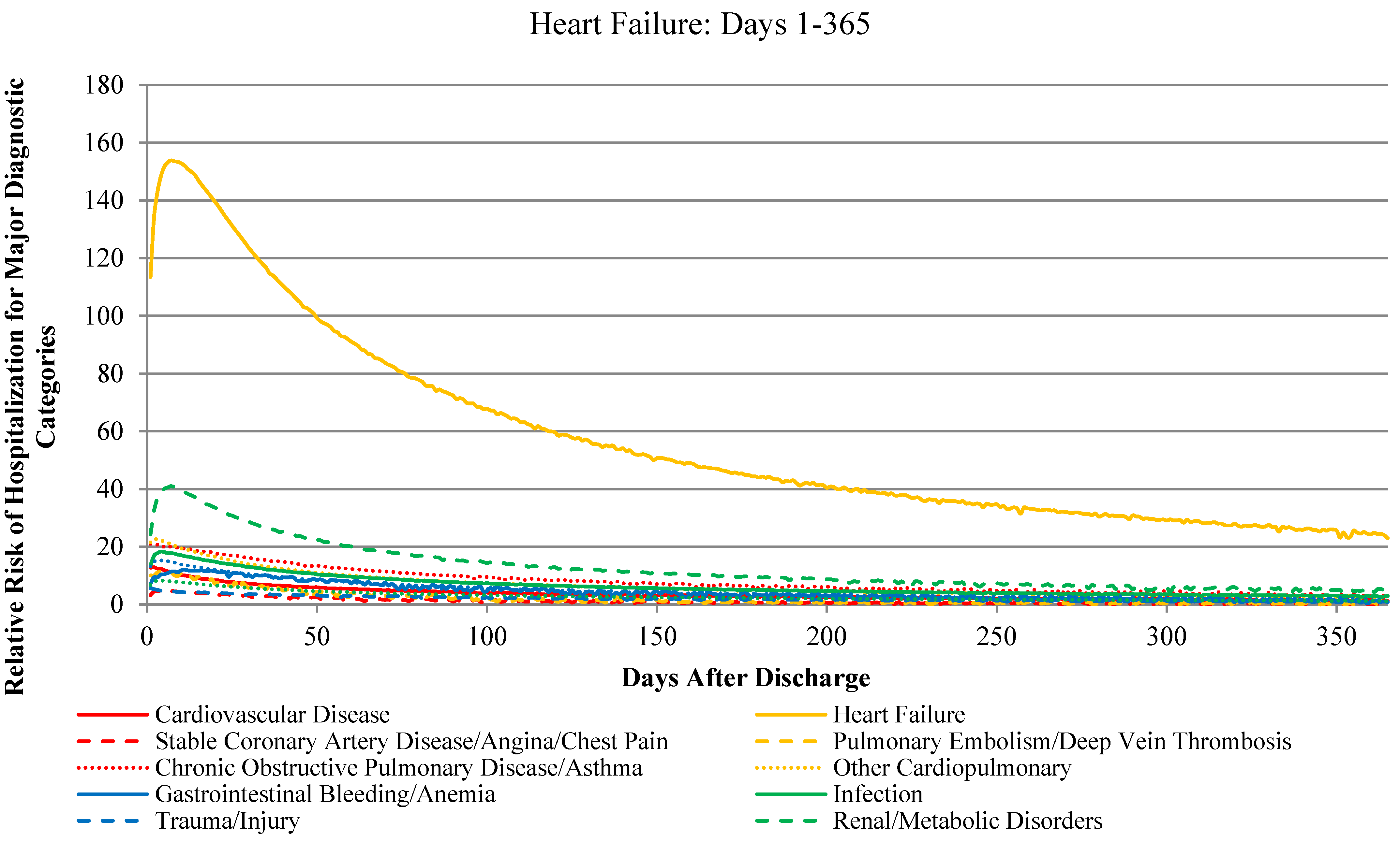

Supplement: S1 Fig — Relative risk information for HF. Each line represents 1 of the 12 readmission diagnostic categories. (TIFF) [file pone.0160492.s001.tiff]

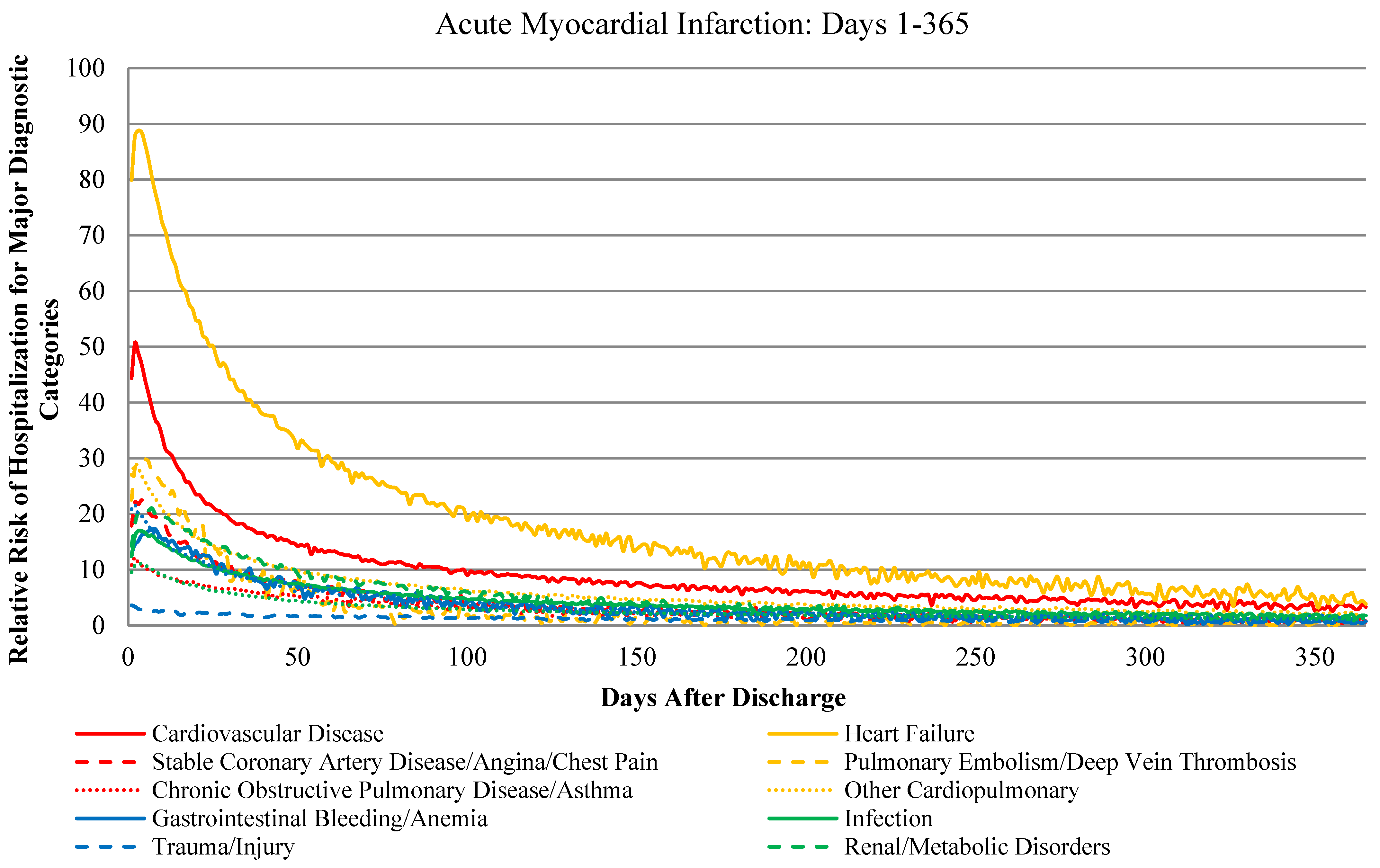

Supplement: S2 Fig — Relative risk information for AMI. Each line represents 1 of the 12 readmission diagnostic categories. (TIF) [file pone.0160492.s002.tif]

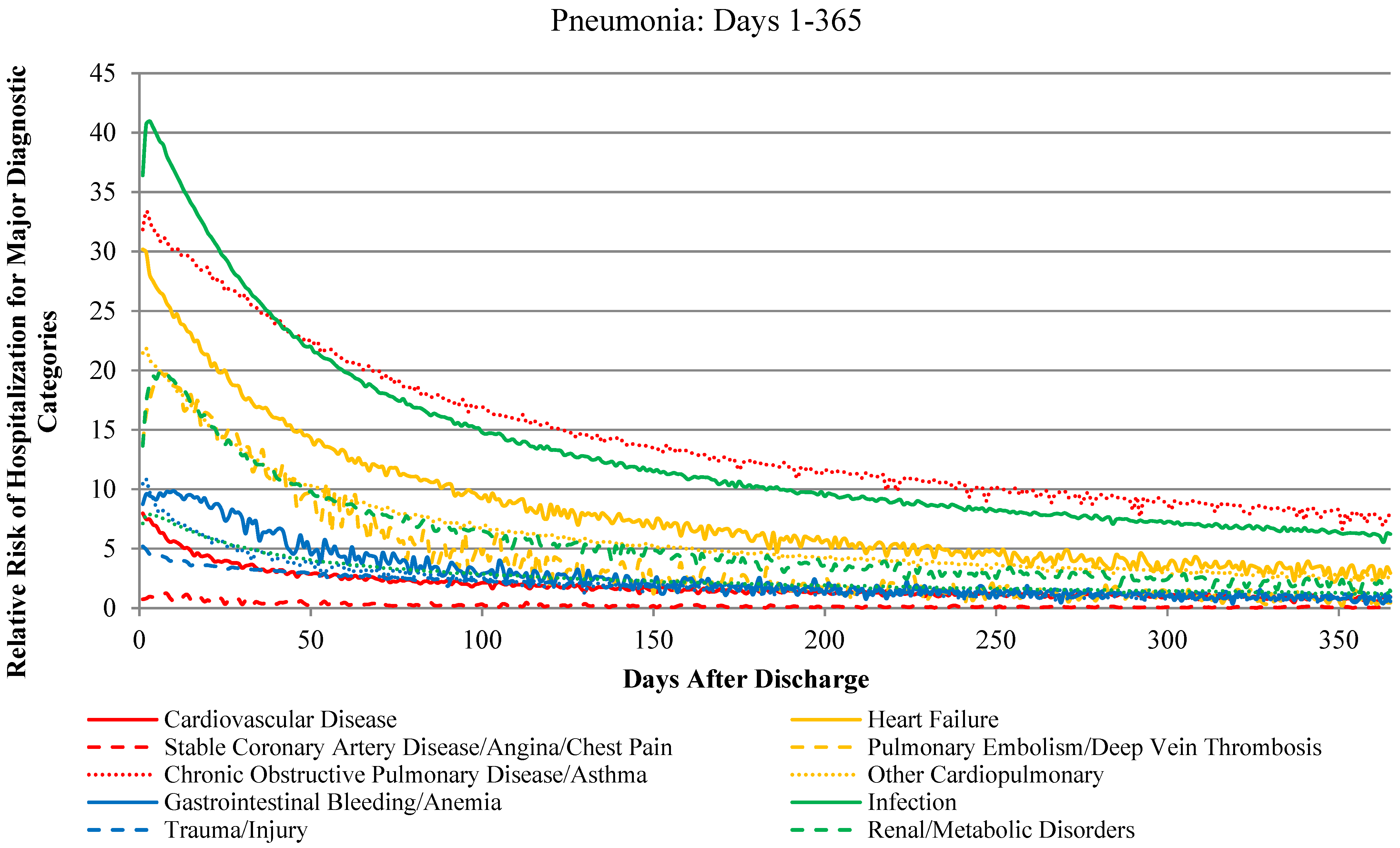

Supplement: S3 Fig — Each line represents 1 of the 12 readmission diagnostic categories. (TIF) [file pone.0160492.s003.tif]
