# Supplementary material for: Trajectories of Risk for Specific Readmission Diagnoses after Hospitalization for Heart Failure, Acute Myocardial Infarction, or Pneumonia
Source: PLoS One. 2016 Oct 7;11(10):e0160492. doi: 10.1371/journal.pone.0160492 (PMC5055318; doi:10.1371/journal.pone.0160492)
Supplement: S1 Table — (DOCX) [file pone.0160492.s004.docx]

**S1 Table. International Classification of Disease, Ninth Revision, Clinical Modification Codes Used to Define HF, AMI, and Pneumonia Cohorts.**

| **Cohort Name** | **Constituent ICD-9-CM Codes** |
| --- | --- |
| Heart Failure | 402.01, 402.11, 402.91, 404.01, 404.03, 404.11, 404.13, 404.91, 404.93, 428.xx |
| Acute Myocardial Infarction | 410.xx excluding those with 410.x2 (AMI, subsequent episode of care) |
| Pneumonia | 480.x, 481, 482.xx, 483.x, 485, 486, 487.0, 488.11 |

ICD-9-CM: International Classification of Diseases, Ninth Revision, Clinical Modification
