# Supplementary material for: Trajectories of Risk for Specific Readmission Diagnoses after Hospitalization for Heart Failure, Acute Myocardial Infarction, or Pneumonia
Source: PLoS One. 2016 Oct 7;11(10):e0160492. doi: 10.1371/journal.pone.0160492 (PMC5055318; doi:10.1371/journal.pone.0160492)
Supplement: S2 Table — (DOCX) [file pone.0160492.s005.docx]

**S2 Table. Readmission Diagnostic Category Constituents.**

| **Readmission Diagnostic Category Number and Name** | **Constituent CMS Condition Category Codes** | **Additional ICD-9-CM Diagnostic Codes** |
| --- | --- | --- |
| 1 – Cardiovascular Disease | 81, 82, 96, 97 |  |
| 2 - Heart Failure | 80 |  |
| 3 - Stable CAD/Angina/Chest Pain | 83, 84 (excluded ICD-9-CM codes on right) | Exclude 786.50, 786.59 |
| 4 – Pulmonary Embolism/Deep Vein Thrombosis | N/A (constituent ICD-9-CM codes on right) | 415.1, 415.11, 415.19, 453.4, 453.40, 453.41, 453.42, 453.8 453.81, 453.82, 453.83, 453.84, 453.85, 453.86, 453.87, 453.89, 453.9 |
| 5 – Chronic Obstructive Pulmonary Disease/Asthma | 108, 110 |  |
| 6 - Other Cardiopulmonary | 77, 78, 79, 109, 86, 85, 87, 88, 94, 89, 90, 91, 114, 104, 105, 115, 106, 162.9, 162.3, 162.5, 162.8, 162.2, 162.4 (additional and excluded ICD-9-CM codes on right) | Include 786.50, 786.59, 780.2  Exclude 415.1, 415.11, 415.19, 453.4, 453.40, 453.41, 453.42, 453.8 453.81, 453.82, 453.83, 453.84, 453.85, 453.86, 453.87, 453.89, 453.9 |
| 7 - Gastrointestinal Bleeding/Anemia | 47, 95 (additional ICD-9-CM codes on right) | 531.0, 531.00, 531.01, 531.2, 531.20, 531.21, 531.40, 531.41, 531.6, 531.60, 531.61, 532.0, 532.00, 532.01, 532.2, 532.20, 532.21, 532.4, 532.40, 532.41, 532.6, 532.60 |
| 8 - Infection | 1, 3, 4, 5, 6, 111, 112, 113, 2, 135, 152 (additional ICD-9-CM codes on right) | 8.45 |
| 9 - Trauma/Injury | 154, 155, 156, 157, 158, 159, 160, 161, 162 (excluded ICD-9-CM codes on right) | Exclude 162.9, 162.3, 162.5, 162.8, 162.2, 162.4 |
| 10 - Renal/Metabolic Disorders | 131, 23, 21, 22, 24, 128, 129, 130, 132, 15, 16, 17, 18, 19, 20 |  |
| 11 - Arrhythmia/Conduction Disorders | 92, 93 |  |
| 12 - Other | See below | See below |
| Cancer | 7, 8, 9, 10, 11, 12, 13, 14 |  |
| Liver Disease | 25, 26, 27, 28, 29 |  |
| Digestive (Gastrointestinal) | 30, 31, 32, 33, 34, 35, 36 (Excluded ICD-9-CM codes on right) | Exclude 531.0, 531.00, 531.01, 531.2, 531.20, 531.21, 531.40, 531.41, 531.6, 531.60, 531.61, 532.0, 532.00, 532.01, 532.2, 532.20, 532.21, 532.4, 532.40, 532.41, 532.6 |
| Musculoskeletal | 37, 38, 39, 40, 41, 42, 43 |  |
| Other Hematologic | 44, 45, 46 |  |
| Cognitive | 48, 49, 50 |  |
| Substance Abuse | 51, 52, 53 |  |
| Psychiatric | 54, 55, 56, 57, 58, 59, 60 |  |
| Development Disorders | 61, 62, 63, 64, 65, 66 |  |
| Spinal Cord | 67, 68, 69 |  |
| Neuromuscular | 70, 71, 72, 73, 74, 75, 76 |  |
| Non-acute Cerebrovascular Disease | 98, 99, 100, 101, 102, 103, 107 |  |
| Ophthalmologic | 116, 117, 118, 119, 120, 121, 122, 123, 124 |  |
| Ear, Nose, and Throat | 125, 126, 127 |  |
| Genitourinary | 133, 134, 136, 137, 138, 139, 140 |  |
| Obstetric | 141, 142, 143, 144, 145, 146, 147 |  |
| Other Dermatologic | 150, 151, 153 |  |
| Symptoms (Major and Minor) Excluding Respiratory | 166, 167 (Excluded ICD-9-CM codes on right) | Exclude 786.0, 786.00, 786.01, 786.02, 786.05, 786.06, 786.50, 786.59, 786.9, 780.2 |
| Neonatal | 168, 169, 170, 171, 172 |  |
| Organ Transplant | 173, 174, 175 |  |
| Miscellaneous Surgical and Non-Surgical Procedures | 176, 177, 178, 179, 180, 181, 182 |  |
| Durable Medical Equipment | 185, 186, 187, 188, 189 |  |
| Other | 183, 184 |  |
| Skin Ulcer Including Decubitus Ulcer | 148, 149 |  |
| Complications of Care | 163, 164, 165 |  |

CMS: Centers for Medicare & Medicaid Services; ICD-9-CM: International Classification of Diseases, Ninth Revision, Clinical Modification
