# Supplementary material for: Trajectories of Risk for Specific Readmission Diagnoses after Hospitalization for Heart Failure, Acute Myocardial Infarction, or Pneumonia
Source: PLoS One. 2016 Oct 7;11(10):e0160492. doi: 10.1371/journal.pone.0160492 (PMC5055318; doi:10.1371/journal.pone.0160492)
Supplement: S3 Table — (DOCX) [file pone.0160492.s006.docx]

**S3 Table. Age, Race, and Sex Distribution for the 3 Condition Cohorts and Comparator Population.**

|  |  | **Heart Failure (n=1,922,580)** | **Acute Myocardial Infarction (n=742,335)** | **Pneumonia (n=1,497,271)** | **Comparator Population (n=27,764,699)** |
| --- | --- | --- | --- | --- | --- |
| **Age Category** |  |  |  |  |  |
|  | 65-74 | 442,185 | 263,647 | 361,513 | 14,608,846 |
|  | 75-84 | 666,173 | 277,550 | 530,478 | 9,142,881 |
|  | ≥85 | 814,222 | 201,138 | 605,280 | 4,012,972 |
| **Race** |  |  |  |  |  |
|  | White | 1,594,721 | 651,880 | 1,328,173 | 24,074,306 |
|  | Black | 240,506 | 57,441 | 100,253 | 2,118,292 |
|  | Other | 87,353 | 33,014 | 68,845 | 1,572,101 |
| **Sex** |  |  |  |  |  |
|  | Female | 1,072,897 | 369,160 | 825,151 | 15,731,675 |
|  | Male | 849,683 | 373,175 | 672,120 | 12,033,024 |
