# Supplementary material for: Trajectories of Risk for Specific Readmission Diagnoses after Hospitalization for Heart Failure, Acute Myocardial Infarction, or Pneumonia
Source: PLoS One. 2016 Oct 7;11(10):e0160492. doi: 10.1371/journal.pone.0160492 (PMC5055318; doi:10.1371/journal.pone.0160492)
Supplement: S4 Table — (DOCX) [file pone.0160492.s007.docx]

**S4 Table. Relative Risks Comparing Study Populations to the Medicare Fee-For-Service Population for All Outcomes and Conditions Over the First 30, 60, 90, 180, and 365 Days After Discharge.**

**A) Heart Failure**

| **Readmission Diagnosis Category** | **Days After Discharge** | | | | |
| --- | --- | --- | --- | --- | --- |
|  | **30**  **(95% CI)** | **60**  **(95% CI)** | **90**  **(95% CI)** | **180**  **(95% CI)** | **365**  **(95% CI)** |
| **Cardiovascular Disease** | 7.3  (7.1, 7.4) | 5.4  (5.3, 5.5) | 4.3  (4.3, 4.4) | 2.6  (2.5, 2.6) | 1.2  (1.2, 1.2) |
| **Heart Failure** | 123.4  (121.7, 125.2) | 91.1  (90.2, 92.1) | 72.4  (71.8, 73.0) | 44.0  (43.7, 44.2) | 23.0  (22.9, 23.1) |
| **Stable Coronary Artery Disease/Angina/ Chest Pain** | 3.0  (2.9, 3.1) | 1.9  (1.8, 1.9) | 1.0  (1.0, 1.1) | 0.7  (0.7, 0.7) | 0.1  (0.0, 0.1) |
| **Pulmonary Embolism/Deep Vein Thrombosis** | 5.7  (5.4, 6.0) | 3.7  (3.6, 3.9) | 1.8  (1.8, 1.9) | 1.0  (0.9, 1.0) | 0.1  (0.1, 0.1) |
| **Chronic Obstructive Pulmonary Disease/Asthma** | 16.1  (15.8, 16.4) | 12.3  (12.1, 12.5) | 9.9  (9.7, 10.0) | 5.3  (5.3, 5.4) | 3.6  (3.6, 3.6) |
| **Other Cardiopulmonary** | 14.0  (13.8, 14.2) | 10.1  (10.0, 10.2) | 7.9  (7.8, 8.0) | 5.0  (4.9, 5.0) | 2.9  (2.9, 2.9) |
| **Gastrointestinal Bleeding/Anemia** | 10.2  (9.8, 10.5) | 7.3  (7.1, 7.5) | 5.9  (5.8, 6.0) | 3.3  (3.2, 3.3) | 0.9  (0.9, 0.9) |
| **Infection** | 13.0  (12.8, 13.1) | 9.6  (9.5, 9.7) | 7.7  (7.6, 7.8) | 4.9  (4.9, 5.0) | 3.0  (2.9, 3.0) |
| **Trauma/Injury** | 3.6  (3.5, 3.7) | 3.1  (3.0, 3.1) | 2.5  (2.5, 2.6) | 1.6  (1.6, 1.6) | 0.9  (0.9, 0.9) |
| **Renal/Metabolic Disorders** | 28.6  (28.1, 29.0) | 20.1  (19.9, 20.3) | 15.7  (15.6, 15.9) | 9.5  (9.4, 9.5) | 4.3  (4.2, 4.3) |
| **Arrhythmia/**  **Conduction Disorders** | 10.5  (10.3, 10.7) | 7.8  (7.7, 7.9) | 6.2  (6.1, 6.3) | 3.1  (3.1, 3.1) | 1.1  (1.1, 1.1) |
| **Other** | 5.8  (5.7, 5.8) | 4.2  (4.2, 4.3) | 3.4  (3.4, 3.5) | 2.2  (2.2, 2.2) | 1.3  (1.3, 1.3) |

**B) Acute Myocardial Infarction**

| **Readmission Diagnosis Category** | **Days After Discharge** | | | | |
| --- | --- | --- | --- | --- | --- |
|  | **30**  **(95% CI)** | **60**  **(95% CI)** | **90**  **(95% CI)** | **180**  **(95% CI)** | **365**  **(95% CI)** |
| **Cardiovascular Disease** | 19.1  (18.7, 19.4) | 13.3  (13.1, 13.5) | 10.5  (10.4, 10.6) | 6.7  (6.7, 6.8) | 3.3  (3.3, 3.4) |
| **Heart Failure** | 44.3  (43.5, 45.1) | 29.4  (29.0, 29.8) | 22.0  (21.8, 22.3) | 11.1  (10.9, 11.2) | 3.9  (3.8, 3.9) |
| **Stable Coronary Artery Disease/Angina/Chest Pain** | 11.0  (10.7, 11.4) | 6.9  (6.7, 7.1) | 4.8  (4.7, 4.9) | 2.6  (2.5, 2.6) | 0.8  (0.7, 0.8) |
| **Pulmonary Embolism/Deep Vein Thrombosis** | 8.1  (7.6, 8.6) | 2.4  (2.2, 2.6) | 1.7  (1.6, 1.8) | 1.2  (1.1, 1.3) | 0.6  (0.5, 0.6) |
| **Chronic Obstructive Pulmonary Disease/Asthma** | 6.5  (6.3, 6.8) | 4.2  (4.1, 4.4) | 3.6  (3.4, 3.7) | 1.9  (1.9, 2.0) | 0.6  (0.5, 0.6) |
| **Other Cardiopulmonary** | 13.0  (12.8, 13.3) | 8.7  (8.6, 8.8) | 6.7  (6.6, 6.8) | 4.2  (4.1, 4.2) | 1.8  (1.8, 1.8) |
| **Gastrointestinal Bleeding/Anemia** | 9.5  (9.1, 10.0) | 5.8  (5.6, 6.0) | 5  (4.8, 5.2) | 1.7  (1.7, 1.8) | 0.8  (0.7, 0.8) |
| **Infection** | 9.5  (9.3, 9.6) | 6.7  (6.6, 6.8) | 5.1  (5.1, 5.2) | 2.9  (2.9, 3.0) | 1.8  (1.8, 1.8) |
| **Trauma/Injury** | 2.2  (2.1, 2.3) | 1.9  (1.8, 1.9) | 1.3  (1.3, 1.4) | 1.3  (1.3, 1.3) | 0.7  (0.7, 0.8) |
| **Renal/Metabolic Disorders** | 12.9  (12.6, 13.3) | 8  (7.9, 8.2) | 6.4  (6.3, 6.5) | 3.9  (3.8, 3.9) | 1.3  (1.3, 1.3) |
| **Arrhythmia/Conduction Disorders** | 9.6  (9.3, 9.9) | 6.2  (6.0, 6.3) | 3.8  (3.7, 3.9) | 2.1  (2.0, 2.2) | 1.5  (1.5, 1.6) |
| **Other** | 5.8  (5.7, 5.9) | 3.9  (3.8, 3.9) | 3.1  (3.0, 3.1) | 1.8  (1.8, 1.8) | 1  (1.0, 1.1) |

**C) Pneumonia**

| **Readmission Diagnosis Category** | **Days After Discharge** | | | | |
| --- | --- | --- | --- | --- | --- |
|  | **30**  **(95% CI)** | **60**  **(95% CI)** | **90**  **(95% CI)** | **180**  **(95% CI)** | **365**  **(95% CI)** |
| **Cardiovascular Disease** | 3.4  (3.3, 3.5) | 2.7  (2.6, 2.7) | 2.3  (2.3, 2.4) | 1.6  (1.6, 1.6) | 1.0  (0.9, 1.0) |
| **Heart Failure** | 17.9  (17.5, 18.2) | 13.1  (12.9, 13.3) | 10.4  (10.3, 10.6) | 6.1  (6.0, 6.1) | 2.9  (2.9, 3.0) |
| **Stable Coronary Artery Disease/Angina/Chest Pain** | 0.3  (0.3, 0.4) | 0.5  (0.4, 0.5) | 0.3  (0.3, 0.3) | 0.1  (0.1, 0.1) | 0.1  (0.1, 0.1) |
| **Pulmonary Embolism/Deep Vein Thrombosis** | 13.3  (12.7, 13.9) | 9.4  (9.1, 9.7) | 3.8  (3.7, 4.0) | 1.8  (1.8, 1.9) | 0.4  (0.4, 0.5) |
| **Chronic Obstructive Pulmonary Disease/Asthma** | 26.1  (25.6, 26.6) | 20.9  (20.6, 21.2) | 17.4  (17.2, 17.7) | 12.4  (12.2, 12.5) | 7.9  (7.9, 8.0) |
| **Other Cardiopulmonary** | 13.3  (13.1, 13.5) | 9.2  (9.1, 9.3) | 7.3  (7.2, 7.4) | 4.6  (4.6, 4.6) | 2.6  (2.5, 2.6) |
| **Gastrointestinal Bleeding/Anemia** | 7.6  (7.3, 7.9) | 4.3  (4.2, 4.5) | 3.2  (3.1, 3.3) | 1.9  (1.9, 2.0) | 0.6  (0.6, 0.6) |
| **Infection** | 27.4  (27.1, 27.7) | 19.9  (19.7, 20.0) | 16.0  (15.9, 16.1) | 10.3  (10.2, 10.3) | 6.2  (6.2, 6.3) |
| **Trauma/Injury** | 3.2  (3.1, 3.3) | 2.8  (2.8, 2.9) | 1.8  (1.8, 1.9) | 1.6  (1.6, 1.7) | 0.9  (0.9, 0.9) |
| **Renal/Metabolic Disorders** | 12.8  (12.6, 13.1) | 8.7  (8.6, 8.8) | 6.9  (6.8, 7.0) | 3.9  (3.8, 3.9) | 1.4  (1.4, 1.4) |
| **Arrhythmia/Conduction Disorders** | 4.9  (4.7, 5.0) | 3.3  (3.2, 3.4) | 2.5  (2.4, 2.6) | 1.7  (1.7, 1.7) | 0.8  (0.7, 0.8) |
| **Other** | 5.2  (5.1, 5.2) | 3.7  (3.7, 3.8) | 3  (3.0, 3.0) | 2.1  (2.0, 2.1) | 1.2  (1.2, 1.2) |
